# Uric Acid or 1-Methyl Uric Acid in the Urinary Bladder Increases Serum Glucose, Insulin, True Triglyceride, and Total Cholesterol Levels in Wistar Rats

**DOI:** 10.1100/tsw.2003.90

**Published:** 2003-10-05

**Authors:** T. Balasubramanian

**Affiliations:** Department of Physiology, Rajah Muthiah Medical College, Annamalai University, Annamalainagar-608 002, India, USA

**Keywords:** caffeine, cocoa, coffee, diabetes mellitus, glucocorticoids, hibernation, hypercholesterolaemia, hyperglycaemia, hyperinsulinaemia, hyperlipidaemia, insulin, leptin, metabolic syndrome, methyl uric acid, methyl xanthines, migratory bird, noninsulin dependent diabetes mellitus (NIDDM), obesity, tea, theobromine, theophylline, uric acid, urinary bladder

## Abstract

In animals deprived of food for a long period, a drop in the fat mass below 5% of the total body mass results in an increase in blood glucocorticoids and uric acid levels, followed by foraging activity. Since the glucocorticoids increase the uric acid excretion, an increase in the level of uric acid in the bladder urine could be the signal for this feeding behaviour and subsequent fat storage. Accumulation of fat is associated with hyperglycaemia, hyperinsulinaemia, hyperlipidaemia, and hypercholesterolaemia as seen in the metabolic syndrome or hibernation. It is hypothesized that uric acid or its structurally related compound, 1-methyl uric acid (one of the metabolites of the methyl xanthines namely caffeine, theophylline, and theobromine present in coffee, tea, cocoa, and some drugs), can act on the urinary bladder mucosa and increases the blood glucose, insulin, triglyceride, and cholesterol levels. In rats, perfusion of the urinary bladder with saturated aqueous solution of uric acid or 1-methyl uric acid results in a significant increase in the serum levels of glucose, insulin, true triglyceride, and total cholesterol in comparison with perfusion of the bladder with distilled water at 20, 40, and 80 min. The uric acid or the 1-methyl uric acid acts on the urinary bladder mucosa and increases the serum glucose, insulin, true triglyceride, and total cholesterol levels.

## INTRODUCTION

The long-term regulation of energy homeostasis, i.e., fat storage, is the most studied and the least understood physiologic process[1,2,3,4,5,6]. In laboratory rats and migratory birds on long-term fasting, a drop in the fat mass below 5% of the total body mass results in a dramatic increase in blood glucocorticoids and uric acid levels, followed by foraging activity and other behavioural responses[7,8,9,10,11]. The increase in plasma glucocorticoid or uric acid level could act as an afferent signal to stimulate food intake and promote energy (fat) storage in these animals. It has been reported that the glucocorticoid acts centrally to increase food intake but peripherally it produces lipolysis. Glucocorticoid excess per se does not cause a dose-dependent increase in food intake or body weight in normal animals[4], so it cannot play any direct role in restarting the foraging activity following long-term food deprivation and subsequent fat storage. The increase in the plasma uric acid level could possibly be the afferent signal to promote fat storage. There are experimental evidences to support this view. The plasma concentration of the hormone leptin is proportional to the adiposity mass[5,6]. It has been reported that serum concentrations of both leptin and uric acid are closely related both in nonobese and obese persons[12,13]. The glucocorticoids also are found to increase the uric acid excretion[14]. If the uric acid in blood is to act as a stimulus for the food intake, the simultaneous glucocorticoid-induced increase in its urinary excretion will produce an opposite response. Hence, the site of action of uric acid could not be by its presence in the blood but in the urine. The possible site of action for uric acid could be the urinary bladder because urine is stored in the bladder for sometime before it is voided. Accumulation of fat is also associated with hyperglycaemia, hyperinsulinaemia, hyperlipidaemia, and hypercholesterolaemia as seen in the metabolic syndrome[15] and in the hibernating animals as a preparation for hibernation[16,17]. If the uric acid in the bladder urine is expected to stimulate fat accumulation, it can also produce an increase in the blood levels of glucose, insulin, triglyceride, and cholesterol.

Hence, in the present study as a first step it is hypothesized that presence of uric acid in the urinary bladder produces increase in serum glucose, insulin, triglyceride, and cholesterol levels. To test this hypothesis, an experimental design was worked out and the present study carried out. It was also proposed to study the effect of the structurally related 1-methyl uric acid (one of the metabolites of methyl xanthines present in coffee, tea, cocoa, and some drugs) in the urinary bladder on serum glucose, insulin, triglyceride, and cholesterol levels.

## MATERIALS AND METHODS

Adult albino rats of Wistar strain of either sex weighing 200–300 g maintained on a standard laboratory diet and water *ad libitum* were used for the present study. The Indian National Science Academy’s guidelines for the care and use of laboratory animals were followed. After keeping them without food for an overnight period of 12 h, the animals were anaesthetized with sodium pentobarbital ip (45 mg/kg body weight). The animal was placed on a rat-dissecting table and the rectal temperature was maintained at 37ºC. A tracheal cannula was inserted to ensure a free air way. The external jugular veins were used to obtain blood samples and to infuse normal saline or anaesthesia, if required. A midline incision of pelvis was used to expose the urinary bladder. Both the ureters near the urinary bladder were cannulated using 26G needle sleeved on to polythene tubing so that the urine from the kidneys was diverted away and was not allowed to enter the urinary bladder. The distal ends of the cut ureters were also similarly cannulated and were used for perfusing the urinary bladder with test solutions. The urethra near the urinary bladder was cannulated using polythene tubing (O.D 2 mm; I.D 1.5mm) as an outlet for the perfusing solutions. The perfusing fluid temperature was maintained at 37ºC. The rate of perfusion was maintained at 0.3ml/min. The incision was closed using crocodile clips.

Thirty albino rats were divided into three groups of ten each. Group I served as the control group perfused with distilled water. Following cannulation, the bladder was perfused with distilled water. The perfusate was analysed for uric acid at 20 min and was found not to contain any uric acid. Blood samples of 0.5 ml each were obtained at 20-, 40-, and 80-min intervals of perfusion for serum analysis of total cholesterol, true triglyceride, glucose, and insulin. After each collection of 0.5 ml of blood sample, an equal volume of normal saline was infused intravenously.

Group II served as the uric acid perfused group. The animals of this group were treated in a similar way as those of control group (Group I) except that the bladder was perfused with the aqueous solution of uric acid (0.005 g/100 ml) instead of distilled water.

Group III served as the 1-methyl uric acid perfused group. The animals of this group were treated in a similar way as those of control group (Group I) except that the bladder was perfused with the aqueous solution of 1-methyl uric acid (0.0024 g/100 ml) instead of distilled water.

## Separation of Serum

The sample of blood was allowed to clot; the clot to retract, and the serum was separated using a refrigerated centrifuge at 3000 rpm for 15 min. The serum was immediately stored at –20ºC in a deep freezer till analysis.

## Reagents and Analysis

Uric acid (SICO, Germany) was dissolved in distilled water to make up a solution of 0.005 g/100 ml. A saturated solution of 1-methyl uric acid (1-MUA) (Sigma, USA) was prepared in boiling distilled water (2.5 mg/100 ml) and cooled to room temperature. The concentration of 1-MUA in the perfusion solution was determined by spectrophotometry at 291.2 nm (UV–visible double beam spectrophotometer, model UVIDEC-650, JASCO, Japan) and by comparing with standard 1-methyl uric acid dissolved in 0.1 *N* sodium hydroxide solution, and the concentration was found to be 0.0024 g/100 ml of solution. The uric acid in the perfusate was determined enzymatically using the diagnostic kit (catalogue No. Autopak uric 6689, Miles India Limited, India) based on the uricase – peroxidase system. The total cholesterol in serum was determined enzymatically using the diagnostic kit (catalogue No. 352-20 and C0284 Sigma, USA) based on the cholesterol esterase – cholesterol oxidase – peroxidase system. The serum true triglyceride was determined enzymatically using the diagnostic kit (catalogue No. 337 – B and G 1394 Sigma, USA) based on the lipoprotein lipase – glycerol kinase – glycerol phosphate oxidase – peroxidase system. The serum glucose was determined enzymatically using the diagnostic kit (catalogue No. 510 – DA, Sigma, USA) based on the glucose oxidase – peroxidase system. The serum estimations of total cholesterol, true triglyceride, and glucose using the diagnostic kits were carried out in an autoanalyser (Model SEAC CH – 100, Miles India Limited, India). The serum insulin was determined by radioimmunoassay using radioimmunoassay kit for insulin supplied by Bhabha Atomic Research Center, India. The estimation was carried out using I-125 gamma counter (Electronics corporation of India Limited, India).

## Statistical Analysis

Values are presented as mean ± SE. Differences between the mean values were assessed by students' t-test. A $p$ value of less than 0.05 is considered to be statistically significant.

## RESULTS

The serum levels of total cholesterol, true triglyceride, glucose, and insulin of the control group (Group I) at 20, 40, and 80 min of perfusion were compared with those of the uric acid perfused group (Group II) or 1-methyl uric acid perfused group (Group III).

Perfusion of the urinary bladder with uric acid solution showed an increase in the serum total cholesterol level on comparison with perfusion of the urinary bladder with distilled water ($p < 0.001$ at 20 min, $p < 0.01$ at 40 min, $p < 0.001$ at 80 min). Perfusion of the urinary bladder with 1-methyl uric acid solution showed an increase in the serum total cholesterol level on comparison with perfusion of the urinary bladder with distilled water ($p < 0.001$ at 20 min, $p < 0.001$ at 40 min, $p < 0.001$ at 80 min) (Table 1).

**TABLE 1**
**Serum Total Cholesterol Levels Following Perfusion of the Urinary Bladder with DW or UA or 1-MUA at 20, 40, and 80 min**

| Perfusion duration | Serum total cholesterol ( mmol l $^{-1}$ ) | | |
|---|---|---|---|
| | DW | UA | 1-MUA |
| 20 min | 0.94 ± 0.06 | 1.36 ± 0.07 † | 1.48 ± 0.04 † |
| 40 min | 0.95 ± 0.06 | 1.27 ± 0.08 ‡ | 1.43 ± 0.05 † |
| 80 min | 0.79 ± 0.02 | 1.22 ± 0.10 † | 1.36 ± 0.04 † |

DW = Distilled water; UA = 0.005% aqueous uric acid solution; 1-MUA = 0.0024% aqueous 1-methyl uric acid solution; values are mean SE, n = 10 in each group; groups compared: DW vs. UA at 20, 40, or 80 min; DW vs. 1-MUA at 20, 40, or 80 min. † $p < 0.001$, ‡ $p < 0.01$.

Perfusion of the urinary bladder with uric acid solution showed an increase in the serum true triglyceride level on comparison with perfusion of the urinary bladder with distilled water ($p < 0.01$ at 20 min, $p < 0.001$ at 40 min, $p < 0.001$ at 80 min). Perfusion of the urinary bladder with 1-methyl uric acid solution showed an increase in the serum true triglyceride level on comparison with perfusion of the urinary bladder with distilled water ($p < 0.02$ at 20 min, $p < 0.01$ at 40 min, $p < 0.001$ at 80 min) (Table 2).

Perfusion of the urinary bladder with uric acid solution showed an increase in the serum glucose level on comparison with perfusion of the urinary bladder with distilled water ($p < 0.01$ at 20 min, $p < 0.001$ at 40 min, $p < 0.001$ at 80 min). Perfusion of the urinary bladder with 1-methyl uric acid solution showed an increase in the serum glucose level on comparison with perfusion of the urinary bladder with distilled water ($p < 0.01$ at 40 min, $p < 0.001$ at 80 min) (Table 3).

Perfusion of the urinary bladder with uric acid solution showed an increase in the serum insulin level on comparison with perfusion of the urinary bladder with distilled water ($p < 0.01$ at 40 min, $p < 0.01$ at 80 min). Perfusion of the urinary bladder with 1-methyl uric acid solution showed an increase in the serum insulin level on comparison with perfusion of the urinary bladder with distilled water ($p < 0.001$ at 40 min, $p < 0.01$ at 80 min) (Table 4).

**TABLE 2**
**Serum True Triglyceride Levels Following Perfusion of the Urinary Bladder with DW or UA or 1-MUA at 20, 40, and 80 min**

| Perfusion duration | Serum true triglyceride ( $mmol\ l^{-1}$ ) | | |
|---|---|---|---|
| | DW | UA | 1-MUA |
| 20 min | 0.33 ± 0.03 | 0.68 ± 0.09 ‡ | 0.48 ± 0.05 + |
| 40 min | 0.40 ± 0.04 | 0.84 ± 0.10 † | 0.62 ± 0.05 ‡ |
| 80 min | 0.26 ± 0.03 | 0.58 ± 0.06 † | 0.59 ± 0.04 † |

DW = Distilled water; UA = 0.005% aqueous uric acid solution; 1-MUA = 0.0024% aqueous 1-methyl uric acid solution; values are mean SE, n = 10 in each group; groups compared: DW vs. UA at 20, 40, or 80 min; DW vs. 1-MUA at 20, 40, or 80 min. ‡ $p < 0.01$, + $p < 0.02$, † $p < 0.001$.

**TABLE 3**
**Serum Glucose Levels Following Perfusion of the Urinary Bladder with DW or UA or 1-MUA at 20, 40, and 80 min**

| Perfusion duration | Serum glucose ( $mmol\ l^{-1}$ ) | | |
|---|---|---|---|
| | DW | UA | 1-MUA |
| 20 min | 5.00 ± 0.18 | 6.07 ± 0.29 ‡ | 5.49 ± 0.17 |
| 40 min | 5.10 ± 0.17 | 6.96 ± 0.26 † | 8.01 ± 0.80 ‡ |
| 80 min | 5.27 ± 0.13 | 7.80 ± 0.44 † | 7.36 ± 0.51 † |

DW = Distilled water; UA = 0.005% aqueous uric acid solution; 1-MUA = 0.0024% aqueous 1-methyl uric acid solution; values are mean SE, n = 10 in each group; groups compared: DW vs. UA at 20, 40, or 80 min; DW vs. 1-MUA at 20, 40, or 80 min. ‡ $p < 0.01$, † $p < 0.001$.

**TABLE 4**
**Serum Insulin Levels Following Perfusion of the Urinary Bladder with DW or UA or 1-MUA at 20, 40, and 80 min**

| Perfusion duration | Serum insulin ( $\mu U\ ml^{-1}$ ) | | |
|---|---|---|---|
| | DW | UA | 1-MUA |
| 20 min | 41 ± 4 | 49 ± 2 | 49 ± 1 |
| 40 min | 42 ± 4 | 62 ± 4 ‡ | 73 ± 3 † |
| 80 min | 32 ± 3 | 51 ± 4 ‡ | 56 ± 6 ‡ |

DW = Distilled water; UA = 0.005% aqueous uric acid solution; 1-MUA = 0.0024% aqueous 1-methyl uric acid solution; values are mean SE, n = 10 in each group; groups compared: DW vs. UA at 20, 40, or 80 min; DW vs. 1-MUA at 20, 40, or 80 min. ‡ $p < 0.01$, † $p < 0.001$.

## DISCUSSION

The metabolic syndrome is a clinical condition in which hyperglycaemia, hyperinsulinaemia, hyperlipidaemia, and hypercholesterolaemia cluster in an individual. The molecular basis of this metabolic syndrome however has not been elucidated so far[15]. The methyl xanthines viz. caffeine, theophylline, and theobromine present in coffee, tea, cocoa, and some drugs are consumed by human beings[18]. The metabolites of these methyl xanthines are the methyl uric acids, structurally related to uric acid[19]. The demonstration that uric acid or 1-methyl uric acid in the urinary bladder increases serum glucose, insulin, true triglyceride, and total cholesterol in the present study throws some light on the clinical condition, the metabolic syndrome. The multiple risk factors such as hyperglycaemia, hyperinsulinaemia, hyperlipidaemia, and hypercholesterolaemia associated with the metabolic syndrome in human beings could be the result of the large intake of the methyl xanthines. However, further studies are needed for confirming this view before extrapolating the results of the present animal study to human beings. Further, in the present study the molecular mechanism responsible for the increase in serum glucose, insulin, true triglyceride, and total cholesterol level following infusion of the urinary bladder with 1-methyl uric acid or uric acid has not been studied. It could possibly be due to a hitherto unknown humoral factor released excessively from the urinary bladder mucosa in response to uric acid or 1-methyl uric acid.

As a response to seasonal changes, many mammalian and avian species increase their body lipid synthesis and storage to sustain hibernation or to fuel migration. Seasonal fattening is associated with hyperglycaemia, hyperinsulinaemia, insulin resistance, and hyperlipidaemia[20] and the findings of the present study could explain the underlying mechanism for this. It has been reported that the blood glucocorticoid level shows an increase in hibernators during the active season, when the animals increase their food intake and accumulate fat[21]. Since the glucocorticoid increases the uric acid excretion[14], the increased uric acid in the bladder urine could be responsible for the observed seasonal changes in the metabolic state of these animals. The present study is only a qualitative study to prove the role of urinary bladder and uric acid or 1-methyl uric acid in the regulation of serum glucose, insulin, true triglyceride, and total cholesterol. Further studies will be necessary to unravel the exact mechanisms responsible for this interaction.

The present study is based on an acute experimental design, which does not permit the study of uric acid or 1-methyl uric acid in the urinary bladder on fat storage (obesity). Studies based on the present findings will help us to understand the physiological basis of the long-term regulation of energy homeostasis (fat storage) in human beings as well as in hibernating animals and migratory birds. The present study also will be helpful for studies on the clinical condition, the metabolic syndrome.

## ACKNOWLEDGEMENTS

I thank and acknowledge the services of Dr. K. Kannan, Professor of Pharmaceutical Technology for his help in the analytical techniques. I also acknowledge my wife, Mrs. Radhika, for her help in the preparation of this manuscript. I thank Mrs. Jeyalakshmi Vivekanandan for going through the manuscript and giving useful suggestions.

## BIOSKETCH

T. Balasubramanian is a Reader in Physiology, Rajah Muthiah Medical College, Annamalai University, Annamalainagar, India. He received his doctorate in Physiology from Annamalai University and is involved in conducting undergraduate and postgraduate courses under the Faculties of Medicine and Dentistry and is involved in research on metabolic syndrome and antiphospholipid syndrome.
